# Supplementary material for: Seminoma and Embryonal Carcinoma Footprints Identified by Analysis of Integrated Genome-Wide Epigenetic and Expression Profiles of Germ Cell Cancer Cell Lines
Source: PLoS One. 2014 Jun 2;9(6):e98330. doi: 10.1371/journal.pone.0098330 (PMC4041891; doi:10.1371/journal.pone.0098330)
Supplement: File S1 — ZIP file containing DMRforPairs output for significant regions. Please start from the html files. (ZIP) [file pone.0098330.s008.zip › figures/33673.pdf]

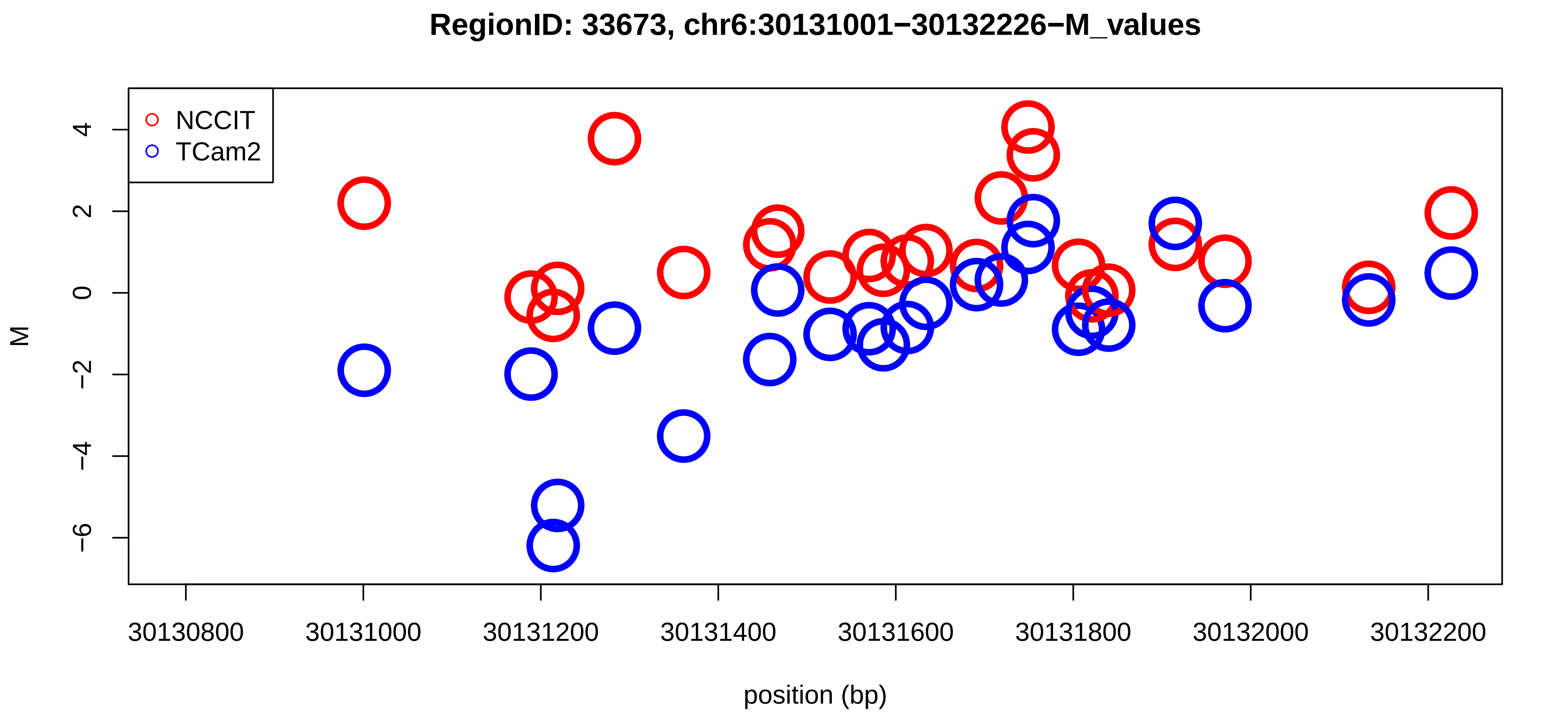
RegionID: 33673, chr6:30131001−30132226−M_values
NCCIT
TCam2
M
4
2
0
−2
−4
−6
30130800
30131000
30131200
30131400
30131600
30131800
30132000
30132200
position (bp)

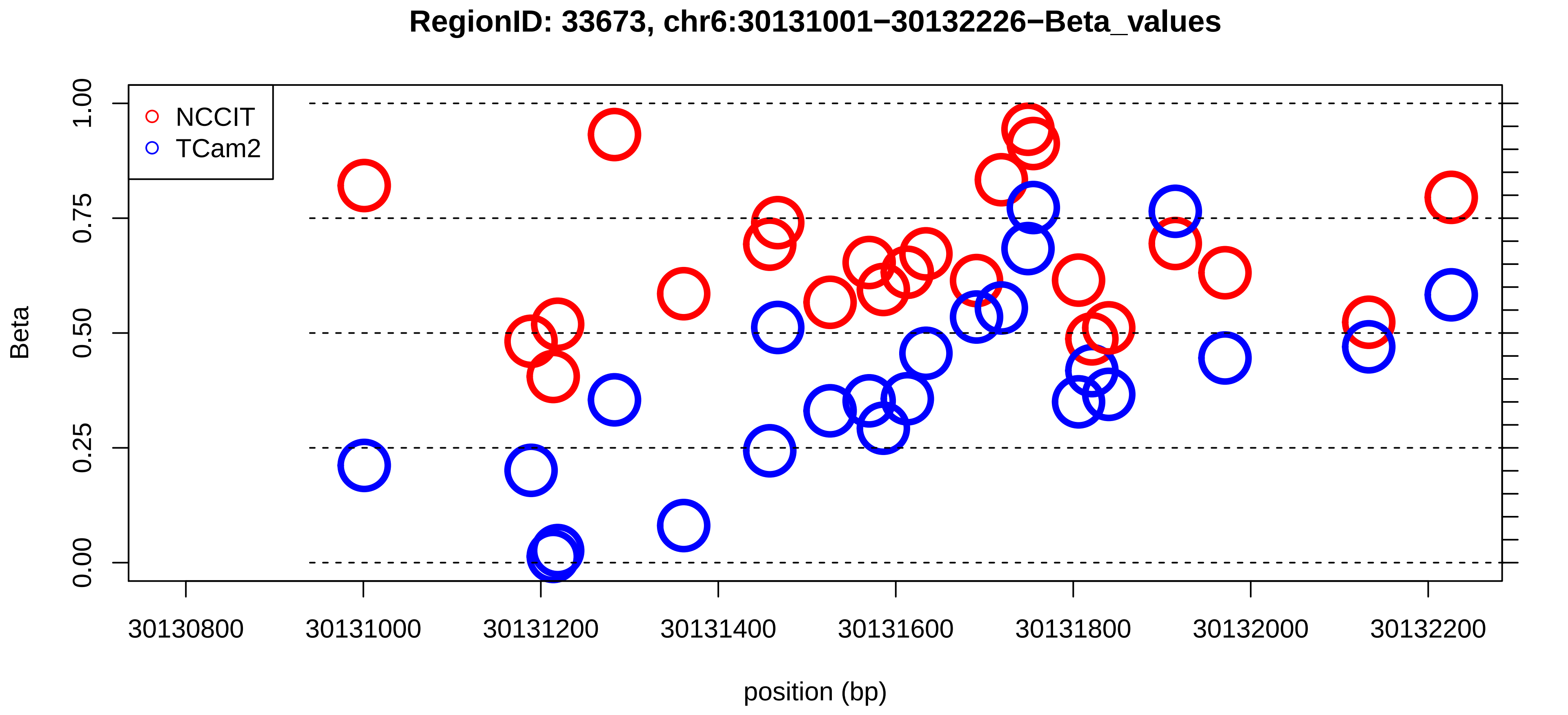
RegionID: 33673, chr6:30131001−30132226−Beta_values
NCCIT
TCam2
Beta
1.00
0.75
0.50
0.25
0.00
30130800
30131000
30131200
30131400
30131600
30131800
30132000
30132200
position (bp)

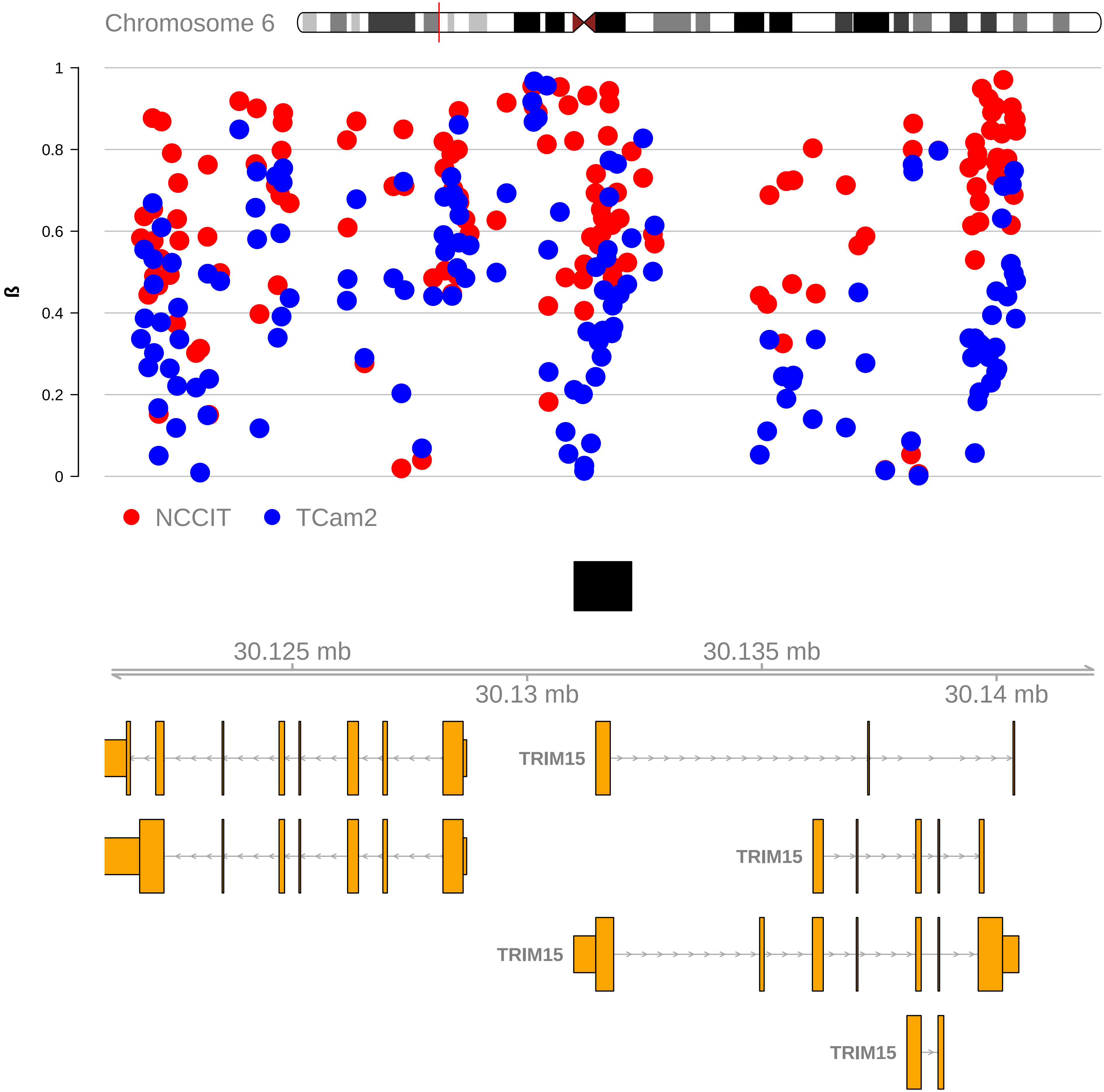
Chromosome 6
ß
1
0.8
0.6
0.4
0.2
0
NCCIT
TCam2
30.125 mb
30.135 mb
30.13 mb
30.14 mb
TRIM15
TRIM15
TRIM15
TRIM15
